# Supplementary material for: Baseline Characterization of the Gut Microbiota of Field and Colony Populations of Phlebotomus tobbi and Preliminary Assessment of the Anti-Leishmanial Activity of Cultivable Bacteria
Source: Pathogens. 2026 Jun 23;15(7):658. doi: 10.3390/pathogens15070658 (PMC13416292; doi:10.3390/pathogens15070658)
Supplement: Supplementary file 1 [file pathogens-15-00658-s001.zip › pathogens-4357168-File S1/Taxa2ASV_Decomposer/Family/f__Xanthomonadaceae/seqs.align.html]

```
seq18           AGTGGGGAATATTGGACAATGGGCGCAAGCCTGATCCAGCCATACCGCGTGGGTGAAGAA
seq48           AGTGGGGAATATTGGACAATGGGCGCAAGCCTGATCCAGCCATACCGCGTGGGTGAAGAA
seq4            AGTGGGGAATATTGGACAATGGGCGCAAGCCTGATCCAGCCATACCGCGTGGGTGAAGAA
seq15           AGTGGGGAATATTGGACAATGGGCGCAAGCCTGATCCAGCCATcCCGCGTGGGTGAAGAA

seq18           GGCCTTCGGGTTGTAAAGCCCTTTTGTTGGGAAAGAAATCCAGCcGGCTAATACCtGGTT
seq48           GGCCTTCGGGTTGTAAAGCCCTTTTGTTGGGAAAGAAATCCAtCTGGCTAATACCCGGgT
seq4            GGCCTTCGGGTTGTAAAGCCCTTTTGTTGGGAAAGAAATCCAGCTGGtTAATACCCGGTT
seq15           GGCCTTCGGGTTGTAAAGCCCTTTTGTTGGGAAAGAAATCCAGCTGGtTAATACCCGGTT

seq18           GGGATGACGGTACCCAAAGAATAAGCACCGGCTAACTTCGTGCCAGCAGCCGCGGTAATA
seq48           GGGATGACGGTACCCAAAGAATAAGCACCGGCTAACTTCGTGCCAGCAGCCGCGGTAATA
seq4            GGGATGACGGTACCCAAAGAATAAGCACCGGCTAACTTCGTGCCAGCAGCCGCGGTAATA
seq15           GGGATGACGGTACCCAAAGAATAAGCACCGGCTAACTTCGTGCCAGCAGCCGCGGTAATA

seq18           CGAAGGGTGCAAGCGTTACTCGGAATTACTGGGCGTAAAGCGTGCGTAGGTGGTCGTTTA
seq48           CGAAGGGTGCAAGCGTTACTCGGAATTACTGGGCGTAAAGCGTGCGTAGGTGGTCGTTTA
seq4            CGAAGGGTGCAAGCGTTACTCGGAATTACTGGGCGTAAAGCGTGCGTAGGTGGTCGTTTA
seq15           CGAAGGGTGCAAGCGTTACTCGGAATTACTGGGCGTAAAGCGTGCGTAGGTGGTCGTTTA

seq18           AGTCCGTTGTGAAAGCCCTGGGCTCAACCTGGGAACTGCAGTGGATACTGGGCGACTAGA
seq48           AGTCCGTTGTGAAAGCCCTGGGCTCAACCTGGGAACTGCAGTGGATACTGGGCGACTAGA
seq4            AGTCCGTTGTGAAAGCCCTGGGCTCAACCTGGGAACTGCAGTGGATACTGGGCGACTAGA
seq15           AGTCCGTTGTGAAAGCCCTGGGCTCAACCTGGGAACTGCAGTGGATACTGGGCGACTAGA

seq18           gTGTGGTAGAGGGTAGCGGAATTCCTGGTGTAGCAGTGAAATGCGTAGAGATCAGGAGGA
seq48           ATGTGGTAGAGGGTAGCGGAATTCCTGGTGTAGCAGTGAAATGCGTAGAGATCAGGAGGA
seq4            ATGTGGTAGAGGGTAGCGGAATTCCTGGTGTAGCAGTGAAATGCGTAGAGATCAGGAGGA
seq15           ATGTGGTAGAGGGTAGCGGAATTCCTGGTGTAGCAGTGAAATGCGTAGAGATCAGGAGGA

seq18           ACATCCATGGCGAAGGCAGCTACCTGGACCAACAcTGACACTGAGGCACGAAAGCGTGGG
seq48           ACATCCATGGCGAAGGCAGCTACCTGGACCAACATTGACACTGAGGCACGAAAGCGTGGG
seq4            ACATCCATGGCGAAGGCAGCTACCTGGACCAACATTGACACTGAGGCACGAAAGCGTGGG
seq15           ACATCCATGGCGAAGGCAGCTACCTGGACCAACATTGACACTGAGGCACGAAAGCGTGGG

seq18           GAGCAAA
seq48           GAGCAAA
seq4            GAGCAAA
seq15           GAGCAAA
```
